# Bis{2-[4-(methyl­sulfan­yl)phen­yl]-1*H*-benzimidazol-3-ium} tetra­bromido­cuprate(II) dihydrate

**DOI:** 10.1107/S1600536811012840

**Published:** 2011-04-13

**Authors:** M. N. Manjunatha, Mohamed Ziaulla, Ravish Sankolli, Noor Shahina Begum, K. R. Nagasundara

**Affiliations:** a Department of Chemistry, Bangalore University, Bangalore 560 001, India; b Solid State and Structural Chemistry Unit, Indian Institute of Science, Bangalore 560 012, India

## Abstract

The asymmetric unit of the title compound, (C_14_H_13_N_2_S)_2_[CuBr_4_]·2H_2_O, contains two cations, one anion and two solvent water mol­ecules that are connected *via* O—H⋯Br, N—H⋯Br and N—H⋯O hydrogen bonds into a two-dimensional polymeric structure. The cations are arranged in a head-to-tail fashion and form stacks along [100]. The central Cu^II^ atom of the anion is in a distorted tetra­hedral environment.

## Related literature

For general background to benzimidazoles and their derivatives, see: Huang & Scarborough *et al.* (1999 ▶); Preston (1974 ▶); Zhu *et al.* (2000 ▶). For related structures, see: Ziaulla *et al.* (2011 ▶).
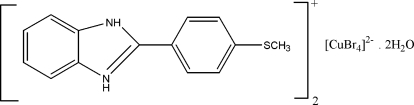


## Experimental

### 

#### Crystal data

(C_14_H_13_N_2_S)_2_[CuBr_4_]·2H_2_O
*M* *_r_* = 901.86
Triclinic, 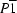

*a* = 7.6878 (5) Å
*b* = 11.8358 (7) Å
*c* = 18.5485 (9) Å
α = 85.305 (4)°
β = 84.778 (5)°
γ = 80.692 (5)°
*V* = 1654.74 (17) Å^3^
*Z* = 2
Mo *K*α radiation
μ = 5.65 mm^−1^
*T* = 296 K
0.18 × 0.16 × 0.16 mm

#### Data collection

Bruker SMART APEX CCD detector diffractometer
Absorption correction: multi-scan (*SADABS*; Bruker, 1998 ▶) *T*_min_ = 0.430, *T*_max_ = 0.465
27134 measured reflections
5805 independent reflections
3344 reflections with *I* > 2σ(*I*)
*R*_int_ = 0.110

#### Refinement

*R*[*F* ^2^ > 2σ(*F* ^2^)] = 0.059
*wR*(*F* ^2^) = 0.127
*S* = 1.00
5805 reflections
384 parameters
6 restraints
H atoms treated by a mixture of independent and constrained refinement
Δρ_max_ = 0.75 e Å^−3^
Δρ_min_ = −0.59 e Å^−3^

### 

Data collection: *SMART* (Bruker, 1998 ▶); cell refinement: *SAINT* (Bruker, 1998 ▶); data reduction: *SAINT*; program(s) used to solve structure: *SHELXS97* (Sheldrick, 2008 ▶); program(s) used to refine structure: *SHELXL97* (Sheldrick, 2008 ▶); molecular graphics: *ORTEP-3 for Windows* (Farrugia, 1997 ▶) and *CAMERON* (Watkin *et al.*, 1996 ▶); software used to prepare material for publication: *WinGX* (Farrugia, 1999 ▶).

## Supplementary Material

Crystal structure: contains datablocks global, I. DOI: 10.1107/S1600536811012840/gk2356sup1.cif

Structure factors: contains datablocks I. DOI: 10.1107/S1600536811012840/gk2356Isup2.hkl

Additional supplementary materials:  crystallographic information; 3D view; checkCIF report

## Figures and Tables

**Table 1:** Hydrogen-bond geometry (Å, °)

*D*—H⋯*A*	*D*—H	H⋯*A*	*D*⋯*A*	*D*—H⋯*A*
N3—H3⋯O1^i^	0.86	1.86	2.703 (8)	165
N2—H2⋯Br3^ii^	0.86	2.44	3.275 (6)	162
O1—H1*D*⋯Br3^iii^	0.85 (6)	2.55 (7)	3.344 (6)	155
O2—H2*A*⋯Br2^iii^	0.83 (4)	2.96 (6)	3.735 (6)	155
O1—H1*E*⋯Br1	0.84 (7)	2.53 (7)	3.359 (6)	170
O2—H2*B*⋯Br4	0.85 (5)	2.77 (7)	3.597 (6)	166

Symmetry codes: (i) 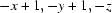; (ii) 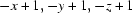; (iii) 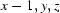.

**Fig. 1.**
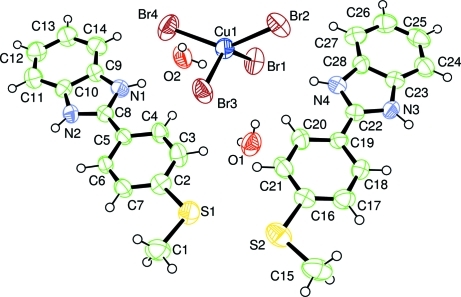
ORTEP (Farrugia, 1997) view of the title compound, showing 50% probability ellipsoids.

**Fig. 2.**
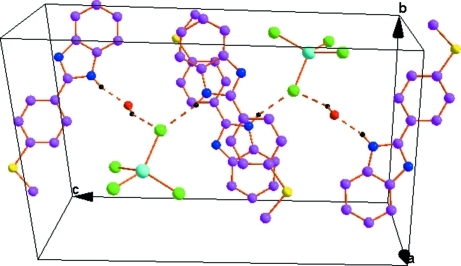
Crystal packing of the title compound showing intermolecular interactions with dotted lines. H-atoms not involved in hydrogen bonding have been excluded.

## supplementary crystallographic information

### Comment

The synthesis of benzimidazoles makes use of solid-phase synthesis *via o*-nitroanilines (Preston *et al.*, 1974; Huang &Scarborough, 1999). Benzimidazole derivatives are effective against the human cytomegalo virus (HCMV) (Zhu *et al.*, 2000). In addition benzimidazole derivatives exhibit a number of important pharmacological properties, such as antihistaminic, anti-ulcerative, antiallergic and antipyretic. In the title compound, as shown in Fig. 1, there are two cations, one tetrabromidocopper(II) anion and two solvent water molecules in the asymmetric unit. The Cu^II^ atom shows strongly distorted tetrahedral geometry, coordinating with four terminal bromine atoms with the bond lengths in the range 2.3389 (1) Å to 2.4084 (1) Å. The Br—Cu—Br bond angles are between 96.18 (4)° and 139.53 (6)°. The benzimidazole and thiomethyl phenyl rings are virtually planar and inclined at an dihedral angle 2.67 (2)° . The bond lengths and angles for the benzimidazole cation of the molecule are in good agreement, within experimental errors, with those observed in other benzimidazole derivatives (Ziaulla *et al.*, 2011). The crystal structure is stabilized by N—H···O, O—H···Br and N—H···Br hydrogen bonds (Fig.2).

### Experimental

An ethanolic solution (15 ml) of the 2-(4-methylsulfanyl phenyl)-1*H*- benzimidazole) (0.960 g, 2 mmol) was added to a solution of copper(II) bromide (0.446 g, 1 mmol) in ethanol (25 ml). The mixture was then treated with 48% HBr (2–3 ml) followed by liquid Br2 (2–3 ml).The mixture was refluxed for nearly six hours during which yellow crystals suitable for X-ray analysis were obtained. The crystals were washed with cold ethanol and dried in vacuum over P_2_O_5_ (yield 1.2 g, 85%).

### Refinement

The H atoms were placed at calculated positions and refined in the riding model approximation with C—H= 0.93-0.96 Å and N—H = 0.86 Å and *U*_iso_(H) = 1.2*U*_eq_(C,N). H atoms of water molecules were refined with restraints imposed on the  O–H and H···H distances [O–H = 0.85 (2) Å, H···H =1.39 (4) Å] and with *U*_iso_(H) = 1.5*U*_eq_(O).

### Crystal data

**Table:**

(C_14_H_13_N_2_S)_2_[CuBr_4_]·2H_2_O	*Z* = 2
*M**_r_* = 901.86	*F*(000) = 886
Triclinic, *P*1	*D*_x_ = 1.810 Mg m^−^^3^
Hall symbol: -P 1	Mo *K*α radiation, λ = 0.71073 Å
*a* = 7.6878 (5) Å	Cell parameters from 5805 reflections
*b* = 11.8358 (7) Å	θ = 2.7–25.0°
*c* = 18.5485 (9) Å	µ = 5.65 mm^−^^1^
α = 85.305 (4)°	*T* = 296 K
β = 84.778 (5)°	Block, yellow
γ = 80.692 (5)°	0.18 × 0.16 × 0.16 mm
*V* = 1654.74 (17) Å^3^	

### Data collection

**Table:**

Bruker SMART APEX CCD detector diffractometer	5805 independent reflections
Radiation source: Enhance (Mo) X-ray Source	3344 reflections with *I* > 2σ(*I*)
graphite	*R*_int_ = 0.110
ω scans	θ_max_ = 25.0°, θ_min_ = 2.7°
Absorption correction: multi-scan (*SADABS*; Bruker, 1998)	*h* = −9→9
*T*_min_ = 0.430, *T*_max_ = 0.465	*k* = −14→14
27134 measured reflections	*l* = −22→22

### Refinement

**Table:**

Refinement on *F*^2^	Primary atom site location: structure-invariant direct methods
Least-squares matrix: full	Secondary atom site location: difference Fourier map
*R*[*F*^2^ > 2σ(*F*^2^)] = 0.059	Hydrogen site location: inferred from neighbouring sites
*wR*(*F*^2^) = 0.127	H atoms treated by a mixture of independent and constrained refinement
*S* = 1.00	*w* = 1/[σ^2^(*F*_o_^2^) + (0.0351*P*)^2^] where *P* = (*F*_o_^2^ + 2*F*_c_^2^)/3
5805 reflections	(Δ/σ)_max_ < 0.001
384 parameters	Δρ_max_ = 0.75 e Å^−^^3^
6 restraints	Δρ_min_ = −0.59 e Å^−^^3^

### Special details

**Table:**

Geometry. All s.u.'s (except the s.u. in the dihedral angle between two l.s. planes) are estimated using the full covariance matrix. The cell s.u.'s are taken into account individually in the estimation of s.u.'s in distances, angles and torsion angles; correlations between s.u.'s in cell parameters are only used when they are defined by crystal symmetry. An approximate (isotropic) treatment of cell s.u.'s is used for estimating s.u.'s involving l.s. planes.
Refinement. Refinement of *F*^2^ against ALL reflections. The weighted *R*-factor *wR* and goodness of fit *S* are based on *F*^2^, conventional *R*-factors *R* are based on *F*, with *F* set to zero for negative *F*^2^. The threshold expression of *F*^2^ > σ(*F*^2^) is used only for calculating *R*-factors(gt) *etc*. and is not relevant to the choice of reflections for refinement. *R*-factors based on *F*^2^ are statistically about twice as large as those based on *F*, and *R*- factors based on ALL data will be even larger.

### Fractional atomic coordinates and isotropic or equivalent isotropic displacement parameters (Å^2^)

**Table:**

	*x*	*y*	*z*	*U*_iso_*/*U*_eq_	
Br3	0.84824 (12)	0.57905 (7)	0.30208 (4)	0.0669 (3)	
Br2	0.87267 (11)	0.78902 (7)	0.15526 (5)	0.0683 (3)	
Cu1	0.66741 (12)	0.74774 (8)	0.25235 (5)	0.0514 (3)	
Br4	0.53451 (13)	0.82526 (8)	0.35966 (5)	0.0790 (3)	
Br1	0.41165 (11)	0.76741 (8)	0.18456 (4)	0.0704 (3)	
S1	0.6325 (3)	0.24760 (18)	0.32773 (11)	0.0633 (6)	
S2	0.9931 (3)	0.15553 (19)	0.17091 (12)	0.0709 (7)	
N1	0.0725 (8)	0.7087 (5)	0.4564 (3)	0.0510 (17)	
H1	0.0834	0.7291	0.4108	0.061*	
N2	0.1042 (7)	0.6081 (5)	0.5582 (3)	0.0455 (15)	
H2	0.1382	0.5532	0.5896	0.055*	
N3	0.6882 (8)	0.5966 (5)	−0.0737 (3)	0.0483 (16)	
H3	0.7331	0.5473	−0.1045	0.058*	
N4	0.6053 (7)	0.6754 (5)	0.0272 (3)	0.0491 (16)	
H4	0.5875	0.6850	0.0729	0.059*	
O1	0.2089 (9)	0.5361 (5)	0.1865 (3)	0.0744 (18)	
O2	0.0818 (8)	0.8287 (6)	0.3236 (3)	0.0755 (17)	
H1E	0.263 (9)	0.593 (5)	0.180 (5)	0.113*	
H1D	0.103 (5)	0.557 (7)	0.204 (5)	0.113*	
H2B	0.188 (5)	0.838 (8)	0.326 (4)	0.113*	
H2A	0.070 (10)	0.811 (8)	0.282 (2)	0.113*	
C1	0.6852 (11)	0.1254 (6)	0.3895 (4)	0.073 (3)	
H1B	0.7592	0.0654	0.3642	0.109*	
H1A	0.5782	0.0988	0.4094	0.109*	
H1C	0.7466	0.1462	0.4280	0.109*	
C2	0.4938 (9)	0.3474 (6)	0.3793 (4)	0.049 (2)	
C3	0.4289 (10)	0.4503 (7)	0.3424 (4)	0.059 (2)	
H3A	0.4626	0.4628	0.2933	0.071*	
C4	0.3167 (9)	0.5329 (6)	0.3772 (4)	0.049 (2)	
H4A	0.2709	0.5996	0.3508	0.059*	
C5	0.2689 (9)	0.5208 (6)	0.4504 (4)	0.0406 (18)	
C6	0.3317 (9)	0.4189 (6)	0.4874 (4)	0.0435 (18)	
H6	0.2973	0.4073	0.5364	0.052*	
C7	0.4443 (10)	0.3339 (6)	0.4531 (4)	0.050 (2)	
H7	0.4878	0.2667	0.4795	0.060*	
C8	0.1509 (9)	0.6090 (6)	0.4875 (4)	0.0418 (18)	
C9	−0.0276 (9)	0.7731 (6)	0.5082 (4)	0.0480 (19)	
C10	−0.0077 (9)	0.7088 (6)	0.5740 (4)	0.0460 (19)	
C11	−0.0876 (10)	0.7504 (7)	0.6385 (4)	0.058 (2)	
H11	−0.0746	0.7078	0.6826	0.070*	
C12	−0.1869 (10)	0.8578 (7)	0.6341 (4)	0.063 (2)	
H12	−0.2439	0.8880	0.6763	0.075*	
C13	−0.2050 (10)	0.9230 (7)	0.5683 (4)	0.064 (2)	
H13	−0.2710	0.9961	0.5678	0.077*	
C14	−0.1274 (10)	0.8812 (6)	0.5045 (4)	0.063 (2)	
H14	−0.1412	0.9239	0.4605	0.076*	
C15	1.0901 (10)	0.0542 (6)	0.1072 (4)	0.077 (3)	
H15C	1.1793	0.0857	0.0758	0.116*	
H15A	1.0005	0.0372	0.0787	0.116*	
H15B	1.1429	−0.0150	0.1326	0.116*	
C16	0.9121 (9)	0.2772 (6)	0.1158 (4)	0.050 (2)	
C17	0.9176 (10)	0.2808 (6)	0.0417 (4)	0.056 (2)	
H17	0.9687	0.2168	0.0171	0.067*	
C18	0.8455 (9)	0.3818 (6)	0.0031 (4)	0.050 (2)	
H18	0.8514	0.3843	−0.0473	0.060*	
C19	0.7664 (9)	0.4771 (6)	0.0380 (4)	0.0432 (18)	
C20	0.7666 (10)	0.4719 (6)	0.1131 (4)	0.056 (2)	
H20	0.7191	0.5363	0.1380	0.067*	
C21	0.8361 (10)	0.3726 (6)	0.1511 (4)	0.061 (2)	
H21	0.8315	0.3701	0.2015	0.074*	
C22	0.6917 (9)	0.5791 (6)	−0.0009 (4)	0.0455 (19)	
C23	0.6011 (9)	0.7062 (6)	−0.0912 (4)	0.0435 (18)	
C24	0.5622 (10)	0.7637 (7)	−0.1566 (4)	0.061 (2)	
H24	0.5965	0.7297	−0.2002	0.073*	
C25	0.4703 (11)	0.8735 (7)	−0.1549 (5)	0.066 (2)	
H25	0.4428	0.9147	−0.1984	0.080*	
C26	0.4178 (10)	0.9240 (7)	−0.0901 (5)	0.064 (2)	
H26	0.3564	0.9985	−0.0911	0.077*	
C27	0.4541 (10)	0.8667 (6)	−0.0245 (4)	0.060 (2)	
H27	0.4172	0.8999	0.0192	0.071*	
C28	0.5485 (10)	0.7572 (6)	−0.0269 (4)	0.050 (2)	

### Atomic displacement parameters (Å^2^)

**Table:**

	*U*^11^	*U*^22^	*U*^33^	*U*^12^	*U*^13^	*U*^23^
Br3	0.0774 (6)	0.0605 (6)	0.0514 (5)	0.0114 (5)	0.0023 (4)	0.0136 (4)
Br2	0.0606 (6)	0.0670 (6)	0.0700 (6)	−0.0054 (4)	0.0039 (5)	0.0200 (5)
Cu1	0.0536 (6)	0.0493 (6)	0.0477 (6)	0.0012 (5)	−0.0026 (5)	−0.0023 (4)
Br4	0.0916 (7)	0.0806 (7)	0.0606 (6)	0.0093 (6)	−0.0038 (5)	−0.0273 (5)
Br1	0.0521 (5)	0.1001 (7)	0.0562 (6)	0.0001 (5)	−0.0059 (4)	−0.0116 (5)
S1	0.0654 (15)	0.0619 (14)	0.0568 (13)	0.0100 (12)	−0.0091 (11)	−0.0053 (11)
S2	0.0672 (15)	0.0632 (15)	0.0771 (16)	−0.0009 (12)	−0.0111 (13)	0.0147 (12)
N1	0.058 (4)	0.042 (4)	0.050 (4)	−0.001 (3)	−0.008 (3)	0.002 (3)
N2	0.049 (4)	0.050 (4)	0.037 (4)	−0.007 (3)	−0.009 (3)	0.001 (3)
N3	0.062 (4)	0.042 (4)	0.039 (4)	−0.001 (3)	−0.002 (3)	−0.008 (3)
N4	0.057 (4)	0.042 (4)	0.047 (4)	−0.008 (3)	−0.004 (3)	0.001 (3)
O1	0.078 (5)	0.078 (4)	0.060 (4)	0.008 (3)	0.003 (4)	−0.017 (3)
O2	0.084 (4)	0.089 (5)	0.054 (4)	−0.021 (4)	−0.011 (3)	0.011 (3)
C1	0.085 (7)	0.051 (5)	0.080 (6)	0.000 (5)	−0.018 (5)	0.000 (5)
C2	0.033 (4)	0.054 (5)	0.059 (5)	0.002 (4)	−0.013 (4)	−0.005 (4)
C3	0.053 (5)	0.072 (6)	0.044 (5)	0.005 (4)	−0.002 (4)	0.005 (4)
C4	0.048 (5)	0.052 (5)	0.041 (5)	0.005 (4)	−0.004 (4)	0.004 (4)
C5	0.045 (4)	0.038 (4)	0.039 (4)	−0.008 (3)	−0.006 (3)	0.004 (3)
C6	0.054 (5)	0.047 (4)	0.032 (4)	−0.017 (4)	−0.004 (4)	0.002 (3)
C7	0.059 (5)	0.047 (5)	0.045 (5)	−0.010 (4)	−0.010 (4)	0.008 (4)
C8	0.039 (4)	0.043 (4)	0.044 (5)	−0.008 (3)	−0.007 (4)	0.000 (3)
C9	0.049 (5)	0.051 (5)	0.042 (5)	−0.008 (4)	0.000 (4)	0.002 (4)
C10	0.045 (5)	0.050 (5)	0.045 (5)	−0.009 (4)	−0.004 (4)	−0.009 (4)
C11	0.052 (5)	0.066 (6)	0.054 (5)	−0.004 (4)	−0.004 (4)	0.001 (4)
C12	0.058 (6)	0.063 (6)	0.063 (6)	−0.002 (5)	0.000 (4)	−0.009 (5)
C13	0.062 (6)	0.055 (5)	0.068 (6)	0.010 (4)	0.001 (5)	−0.006 (5)
C14	0.070 (6)	0.048 (5)	0.067 (6)	0.006 (4)	−0.015 (5)	0.002 (4)
C15	0.063 (6)	0.051 (5)	0.112 (7)	−0.001 (4)	0.003 (5)	0.006 (5)
C16	0.047 (5)	0.047 (5)	0.056 (5)	−0.008 (4)	−0.005 (4)	0.003 (4)
C17	0.055 (5)	0.045 (5)	0.065 (6)	−0.002 (4)	0.000 (4)	−0.007 (4)
C18	0.060 (5)	0.052 (5)	0.040 (4)	−0.010 (4)	−0.007 (4)	−0.007 (4)
C19	0.048 (5)	0.033 (4)	0.047 (5)	−0.004 (3)	0.001 (4)	−0.004 (3)
C20	0.073 (6)	0.053 (5)	0.041 (5)	−0.005 (4)	−0.008 (4)	0.002 (4)
C21	0.078 (6)	0.053 (5)	0.048 (5)	−0.005 (5)	0.001 (4)	0.010 (4)
C22	0.049 (5)	0.041 (4)	0.048 (5)	−0.014 (4)	−0.003 (4)	−0.003 (4)
C23	0.039 (4)	0.042 (4)	0.051 (5)	−0.007 (4)	−0.006 (4)	0.000 (4)
C24	0.069 (6)	0.064 (6)	0.052 (5)	−0.021 (5)	−0.011 (4)	0.008 (4)
C25	0.077 (6)	0.061 (6)	0.067 (6)	−0.025 (5)	−0.034 (5)	0.022 (5)
C26	0.063 (6)	0.047 (5)	0.079 (7)	0.000 (4)	−0.020 (5)	0.004 (5)
C27	0.063 (6)	0.047 (5)	0.069 (6)	−0.005 (4)	−0.009 (5)	−0.008 (4)
C28	0.062 (5)	0.046 (5)	0.039 (5)	−0.003 (4)	−0.007 (4)	0.000 (4)

### Geometric parameters (Å, °)

**Table:**

Br3—Cu1	2.4091 (11)	C6—H6	0.9300
Br2—Cu1	2.3579 (12)	C7—H7	0.9300
Cu1—Br4	2.3396 (12)	C9—C14	1.382 (9)
Cu1—Br1	2.3990 (12)	C9—C10	1.391 (9)
S1—C2	1.743 (7)	C10—C11	1.385 (9)
S1—C1	1.787 (7)	C11—C12	1.373 (9)
S2—C16	1.758 (7)	C11—H11	0.9300
S2—C15	1.782 (7)	C12—C13	1.395 (10)
N1—C8	1.346 (8)	C12—H12	0.9300
N1—C9	1.376 (8)	C13—C14	1.368 (9)
N1—H1	0.8600	C13—H13	0.9300
N2—C8	1.327 (8)	C14—H14	0.9300
N2—C10	1.388 (8)	C15—H15C	0.9600
N2—H2	0.8600	C15—H15A	0.9600
N3—C22	1.351 (8)	C15—H15B	0.9600
N3—C23	1.388 (8)	C16—C17	1.370 (9)
N3—H3	0.8600	C16—C21	1.373 (9)
N4—C22	1.342 (8)	C17—C18	1.402 (9)
N4—C28	1.386 (8)	C17—H17	0.9300
N4—H4	0.8600	C18—C19	1.373 (8)
O1—H1E	0.84 (7)	C18—H18	0.9300
O1—H1D	0.85 (6)	C19—C20	1.390 (9)
O2—H2B	0.85 (5)	C19—C22	1.421 (9)
O2—H2A	0.84 (2)	C20—C21	1.376 (9)
C1—H1B	0.9600	C20—H20	0.9300
C1—H1A	0.9600	C21—H21	0.9300
C1—H1C	0.9600	C23—C24	1.374 (9)
C2—C7	1.390 (9)	C23—C28	1.379 (9)
C2—C3	1.391 (9)	C24—C25	1.377 (10)
C3—C4	1.358 (9)	C24—H24	0.9300
C3—H3A	0.9300	C25—C26	1.385 (10)
C4—C5	1.376 (9)	C25—H25	0.9300
C4—H4A	0.9300	C26—C27	1.373 (10)
C5—C6	1.377 (8)	C26—H26	0.9300
C5—C8	1.443 (9)	C27—C28	1.381 (9)
C6—C7	1.374 (9)	C27—H27	0.9300
			
Br4—Cu1—Br2	139.54 (6)	C12—C11—H11	121.7
Br4—Cu1—Br1	99.30 (4)	C10—C11—H11	121.7
Br2—Cu1—Br1	97.76 (4)	C11—C12—C13	122.0 (7)
Br4—Cu1—Br3	99.91 (4)	C11—C12—H12	119.0
Br2—Cu1—Br3	96.13 (4)	C13—C12—H12	119.0
Br1—Cu1—Br3	130.74 (5)	C14—C13—C12	121.2 (7)
C2—S1—C1	104.8 (4)	C14—C13—H13	119.4
C16—S2—C15	103.5 (4)	C12—C13—H13	119.4
C8—N1—C9	109.9 (6)	C13—C14—C9	117.2 (7)
C8—N1—H1	125.0	C13—C14—H14	121.4
C9—N1—H1	125.0	C9—C14—H14	121.4
C8—N2—C10	109.9 (6)	S2—C15—H15C	109.5
C8—N2—H2	125.0	S2—C15—H15A	109.5
C10—N2—H2	125.0	H15C—C15—H15A	109.5
C22—N3—C23	109.6 (6)	S2—C15—H15B	109.5
C22—N3—H3	125.2	H15C—C15—H15B	109.5
C23—N3—H3	125.2	H15A—C15—H15B	109.5
C22—N4—C28	111.2 (6)	C17—C16—C21	119.4 (7)
C22—N4—H4	124.4	C17—C16—S2	124.3 (6)
C28—N4—H4	124.4	C21—C16—S2	116.3 (6)
H1E—O1—H1D	110 (5)	C16—C17—C18	119.5 (7)
H2B—O2—H2A	109 (5)	C16—C17—H17	120.2
S1—C1—H1B	109.5	C18—C17—H17	120.2
S1—C1—H1A	109.5	C19—C18—C17	121.6 (7)
H1B—C1—H1A	109.5	C19—C18—H18	119.2
S1—C1—H1C	109.5	C17—C18—H18	119.2
H1B—C1—H1C	109.5	C18—C19—C20	117.7 (6)
H1A—C1—H1C	109.5	C18—C19—C22	121.6 (7)
C7—C2—C3	117.7 (6)	C20—C19—C22	120.7 (6)
C7—C2—S1	126.4 (6)	C21—C20—C19	120.8 (7)
C3—C2—S1	116.0 (6)	C21—C20—H20	119.6
C4—C3—C2	120.7 (7)	C19—C20—H20	119.6
C4—C3—H3A	119.7	C16—C21—C20	121.0 (7)
C2—C3—H3A	119.7	C16—C21—H21	119.5
C3—C4—C5	121.9 (6)	C20—C21—H21	119.5
C3—C4—H4A	119.0	N4—C22—N3	106.5 (6)
C5—C4—H4A	119.0	N4—C22—C19	126.9 (7)
C4—C5—C6	117.7 (6)	N3—C22—C19	126.5 (6)
C4—C5—C8	121.9 (6)	C24—C23—C28	120.8 (7)
C6—C5—C8	120.3 (6)	C24—C23—N3	132.0 (7)
C7—C6—C5	121.2 (6)	C28—C23—N3	107.2 (6)
C7—C6—H6	119.4	C23—C24—C25	117.3 (7)
C5—C6—H6	119.4	C23—C24—H24	121.4
C6—C7—C2	120.7 (6)	C25—C24—H24	121.4
C6—C7—H7	119.7	C24—C25—C26	121.6 (7)
C2—C7—H7	119.7	C24—C25—H25	119.2
N2—C8—N1	107.8 (6)	C26—C25—H25	119.2
N2—C8—C5	126.5 (6)	C27—C26—C25	121.5 (7)
N1—C8—C5	125.7 (6)	C27—C26—H26	119.2
N1—C9—C14	132.3 (7)	C25—C26—H26	119.2
N1—C9—C10	106.2 (6)	C26—C27—C28	116.4 (7)
C14—C9—C10	121.5 (7)	C26—C27—H27	121.8
C11—C10—N2	132.5 (7)	C28—C27—H27	121.8
C11—C10—C9	121.3 (7)	C23—C28—C27	122.4 (7)
N2—C10—C9	106.1 (6)	C23—C28—N4	105.4 (6)
C12—C11—C10	116.7 (7)	C27—C28—N4	132.1 (7)
			
C1—S1—C2—C7	2.6 (8)	C15—S2—C16—C17	4.3 (8)
C1—S1—C2—C3	−178.0 (6)	C15—S2—C16—C21	−177.1 (6)
C7—C2—C3—C4	−2.0 (12)	C21—C16—C17—C18	0.0 (12)
S1—C2—C3—C4	178.6 (6)	S2—C16—C17—C18	178.6 (6)
C2—C3—C4—C5	2.9 (12)	C16—C17—C18—C19	−1.1 (12)
C3—C4—C5—C6	−3.2 (11)	C17—C18—C19—C20	2.6 (12)
C3—C4—C5—C8	179.4 (7)	C17—C18—C19—C22	−179.4 (7)
C4—C5—C6—C7	2.7 (11)	C18—C19—C20—C21	−3.1 (12)
C8—C5—C6—C7	−179.9 (7)	C22—C19—C20—C21	178.9 (8)
C5—C6—C7—C2	−1.9 (11)	C17—C16—C21—C20	−0.5 (13)
C3—C2—C7—C6	1.5 (11)	S2—C16—C21—C20	−179.2 (7)
S1—C2—C7—C6	−179.1 (6)	C19—C20—C21—C16	2.1 (13)
C10—N2—C8—N1	0.3 (8)	C28—N4—C22—N3	−1.4 (8)
C10—N2—C8—C5	178.6 (7)	C28—N4—C22—C19	−179.2 (7)
C9—N1—C8—N2	−0.3 (8)	C23—N3—C22—N4	1.1 (8)
C9—N1—C8—C5	−178.6 (7)	C23—N3—C22—C19	178.9 (7)
C4—C5—C8—N2	−176.3 (7)	C18—C19—C22—N4	177.1 (7)
C6—C5—C8—N2	6.4 (11)	C20—C19—C22—N4	−4.9 (12)
C4—C5—C8—N1	1.7 (12)	C18—C19—C22—N3	−0.2 (12)
C6—C5—C8—N1	−175.6 (7)	C20—C19—C22—N3	177.7 (7)
C8—N1—C9—C14	177.7 (8)	C22—N3—C23—C24	−179.4 (8)
C8—N1—C9—C10	0.2 (8)	C22—N3—C23—C28	−0.4 (8)
C8—N2—C10—C11	−177.9 (8)	C28—C23—C24—C25	0.4 (12)
C8—N2—C10—C9	−0.1 (8)	N3—C23—C24—C25	179.2 (8)
N1—C9—C10—C11	178.0 (7)	C23—C24—C25—C26	−0.5 (13)
C14—C9—C10—C11	0.2 (12)	C24—C25—C26—C27	−0.4 (14)
N1—C9—C10—N2	−0.1 (8)	C25—C26—C27—C28	1.2 (13)
C14—C9—C10—N2	−177.9 (7)	C24—C23—C28—C27	0.5 (12)
N2—C10—C11—C12	177.5 (8)	N3—C23—C28—C27	−178.6 (7)
C9—C10—C11—C12	0.1 (12)	C24—C23—C28—N4	178.7 (7)
C10—C11—C12—C13	−0.9 (12)	N3—C23—C28—N4	−0.4 (8)
C11—C12—C13—C14	1.6 (14)	C26—C27—C28—C23	−1.3 (12)
C12—C13—C14—C9	−1.3 (13)	C26—C27—C28—N4	−178.9 (8)
N1—C9—C14—C13	−176.7 (8)	C22—N4—C28—C23	1.1 (8)
C10—C9—C14—C13	0.4 (12)	C22—N4—C28—C27	179.1 (8)

### Hydrogen-bond geometry (Å, °)

**Table:**

*D*—H···*A*	*D*—H	H···*A*	*D*···*A*	*D*—H···*A*
C13—H13···Br4^i^	0.93	2.99	3.831 (8)	150
N3—H3···O1^ii^	0.86	1.86	2.703 (8)	165
C18—H18···O1^ii^	0.93	2.73	3.614 (9)	159
N2—H2···Br3^iii^	0.86	2.44	3.275 (6)	162
O1—H1D···Br3^iv^	0.85 (6)	2.55 (7)	3.344 (6)	155
O2—H2A···Br2^iv^	0.83 (4)	2.96 (6)	3.735 (6)	155
O1—H1E···Br1	0.84 (7)	2.53 (7)	3.359 (6)	170
O2—H2B···Br4	0.85 (5)	2.77 (7)	3.597 (6)	166

Symmetry codes: (i) −*x*, −*y*+2, −*z*+1; (ii) −*x*+1, −*y*+1, −*z*; (iii) −*x*+1, −*y*+1, −*z*+1; (iv) *x*−1, *y*, *z*.
